# The pathobiology of select adolescent young adult lymphomas

**DOI:** 10.1002/jha2.785

**Published:** 2023-09-29

**Authors:** Christian Steidl, Robert Kridel, Michael Binkley, Lindsay M. Morton, Amy Chadburn

**Affiliations:** ^1^ Centre for Lymphoid Cancer BC Cancer Vancouver British Columbia Canada; ^2^ Princess Margaret Cancer Centre ‐ University Health Network Toronto Ontario Canada; ^3^ Department of Radiation Oncology Stanford University Stanford California USA; ^4^ Radiation Epidemiology Branch Division of Cancer Epidemiology and Genetics National Cancer Institute Rockville Maryland USA; ^5^ Department of Pathology and Laboratory Medicine Weill Cornell Medicine New York New York USA

**Keywords:** adolescent young adult lymphomas, aggressive B‐cell lymphomas, pathobiology

## Abstract

Lymphoid cancers are among the most frequent cancers diagnosed in adolescents and young adults (AYA), ranging from approximately 30%–35% of cancer diagnoses in adolescent patients (age 10–19) to approximately 10% in patients aged 30–39 years. Moreover, the specific distribution of lymphoid cancer types varies by age with substantial shifts in the subtype distributions between pediatric, AYA, adult, and older adult patients. Currently, biology studies specific to AYA lymphomas are rare and therefore insight into age‐related pathogenesis is incomplete. This review focuses on the paradigmatic epidemiology and pathogenesis of select lymphomas, occurring in the AYA patient population. With the example of posttransplant lymphoproliferative disorders, nodular lymphocyte‐predominant Hodgkin lymphoma, follicular lymphoma (incl. pediatric‐type follicular lymphoma), and mediastinal lymphomas (incl. classic Hodgkin lymphoma, primary mediastinal large B cell lymphoma and mediastinal gray zone lymphoma), we here illustrate the current state‐of‐the‐art in lymphoma classification, recent molecular insights including genomics, and translational opportunities. To improve outcome and quality of life, international collaboration in consortia dedicated to AYA lymphoma is needed to overcome challenges related to siloed biospecimens and data collections as well as to develop studies designed specifically for this unique population.

## 1 INTRODUCTION

It has long been recognized that the epidemiology of lymphoid malignancies among the Adolescent Young Adult (AYA) population differs from that of other age groups. Based on population‐based cancer registry data from the US National Cancer Institute Surveillance, Epidemiology, and End Results (SEER) Program covering approximately one‐quarter of the US population [1], lymphoid malignancies account for a substantial fraction of total cancers diagnosed among AYA, ranging from approximately one‐third of cancer diagnoses in adolescents to 10% in patients aged 30–39 years. The distribution of specific lymphoid malignancy types also is distinct by age, shifting from a predominance of precursor leukemia/lymphoma and Hodgkin lymphoma (HL) in adolescents to a predominance of diffuse large B‐cell lymphoma (DLBCL) and HL in patients aged 30–39 years. Further analyses demonstrate that incidence as well as survival patterns differ not only by age but also by sex and race/ethnicity [2]. The factors that underlie these differences are not known but may relate to a combination of genetic factors, prevalence of key risk factors (such as infection with human immunodeficiency virus or hepatitis C virus), or social determinants. Population studies will continue to provide valuable tools for understanding the disease burden among AYA and contribute to furthering understanding of how disease biology and patient outcomes may differ compared with patients at both the younger and older extremes of the age spectrum.

In June 2022, physicians and scientists from more than 40 academic and medical institutions, federal agencies and pharmaceutical companies met in Jersey City, New Jersey, for the second Lymphoma Research Foundation (LRF) Adolescent and Young Adult (AYA) Lymphoma Consortium meeting. The overarching goal of the meeting was to advance treatment, care, and long‐term survival for the AYA patient population and shape a vibrant community for collaboration and community engagement. One of the objectives in this process is the collection of biological insights and formulation of future plans to address knowledge gaps and obstacles in clinical translation related to epidemiology and disease biology. In this review we will summarize meeting content and illustrate recent progress in select lymphoma types, that despite being rare, deserve specific attention to improve outcomes in this understudied and clinically underserved patient population.

## 2 POSTTRANSPLANT LYMPHOPROLIFERATIVE DISORDERS

Post‐transplant lymphoproliferative disorders (PTLDs) are a well‐known complication of solid organ and stem cell transplantation. There are a number of factors associated with their development including the type of organ transplanted, younger patient age and negative Epstein Barr virus (EBV) status at the time of transplant [3, 4, 5, 6, 7]. Understanding the biology of PTLDs arising in younger patients is difficult since in the literature the younger patients are often grouped with adult PTLD patients or, due to numbers, are studied as a group over a long period of time, during which immunosuppression protocols, PTLD treatment as well as PTLD diagnostic criteria and testing have changed. However, as approximately 6000 of the 43,000 organ transplants performed in the United States in 2022 were in patients less than 34 years of age (https://unos.org) and as PTLDs account for >85% of the malignancies in the post‐transplant pediatric patient population [8], our need to understand PTLDs in the younger population is imperative. In this discussion we will focus on solid organ transplant (SOT)‐related PTLDs.

Two of the more important risk factors for developing SOT PTLD are age and EBV status at the time of transplantation. However, these two risk factors are intertwined as nearly all adults (>90%) but only ∼45% of pediatric patients (<18 years) and ∼25% of children (<5 years) in western countries are EBV seropositive [9]. A number of studies in SOT recipients have shown that recipient EBV seronegativity and recipient / donor EBV status mismatch at transplant are highly associated with the development of PTLD [8, 10, 11, 12]. The younger transplant recipients, who are more often EBV negative, are thought to be at a higher risk for PTLD due to a lack of an EBV‐directed cytotoxic T cell population from a previous infection as well as an inability to mount a sufficient immune response secondary to the immunosuppression needed for graft retention. Thus, the younger SOT recipients are thought to be unable to recognize and suppress EBV‐induced lymphoid proliferations, that is, PTLDs [7].

The type of solid organ transplanted is an important risk factor and impacts the overall incidence of PTLD in adults and in younger patients. Kidney transplants, which are associated with a very low incidence of PTLD (∼1.0% in adults), accounted for the majority (over 60%) of the SOT in adults in 2022, while the combined number of liver and heart transplants, associated with higher risks of developing PTLD (2.0‐13.2% in children), accounted for approximately the same percentage of transplants in children (https://unos.org) [3, 7, 13, 14, 15, 16, 17]. These differences in risk for the various types of organs transplanted are likely due to a number of factors, including the level of immunosuppression for graft retention [3, 4, 18].

The lymphoid proliferations arising in the post‐transplant setting are divided into different categories based primarily on morphology and molecular‐genetic features [19, 20, 21, 22]. The classification of these lesions, compared to the 2017 WHO and the International Consensus Classification (ICC), is somewhat changed in the upcoming 2023 WHO classification [22, 23, 24]. The relative proportion of these different PTLD subtypes arising in younger patients is different than what is seen in adults (Table 1). Lesions classified as hyperplasia are extremely rare in adults, but account for approximately 20%–30% of PTLDs in the younger SOT recipients. In contrast, adult SOT recipients are more frequently diagnosed with PTLD lymphoma in comparison to younger patients [11, 12, 25, 26]. The location of disease is also somewhat different. Although both adults and younger patients tend to develop disease in extranodal sites, particularly the gastrointestinal tract, there is a predilection for disease to present in the head and neck region, particularly in the tonsils, adenoids and/or cervical lymph nodes in younger patients [11, 12, 25, 26, 27, 28, 29, 30, 31, 32, 33, 34, 35, 36], a pattern of disease that is suggestive of a primary EBV infection [11, 12, 25, 26, 28, 29, 31, 32, 33, 34, 36]. In contrast, PTLDs occurring in adults are more often EBV negative and tend to be more often negative the farther out from transplantation they occur [32, 33, 34]. The most common type of PTLD‐associated lymphoma in younger patients (as in adults) is diffuse large B cell lymphoma accounting for ∼60% of cases, with Burkitt lymphoma accounting for another 8%–10% [37].

**TABLE 1:** Comparison of solid organ transplant (SOT) PTLDs in pediatric and adult patients^*^.

	Pediatric SOT	Adult SOT
**Histology** ^**^		
Hyperplasia/Non‐destructive	31%	2.5%
Polymorphic	41%	22%
Lymphoma/Monomorphic	26%	74%
cHL	3%	1%
**Location**		
Nodal	56%	65%
Extranodal	44%	35%
Common sites†	1. Tonsil/Adenoids 2. Gastrointestinal tract	1. Lymph nodes 2. Gastrointestinal tract
**Miscellaneous**		
EBV (EBER)+	74%	55%
Time TXP → PTLD	27 mo. (median)	43 mo. (median)

Abbreviations: cHL, classic Hodgkin lymphoma‐like; EBER, Epstein‐Barr virus‐encoded small RNAs; EBV, Epstein‐Barr Virus; mo, months; TXP, transplantation.

* Ref: 36 except for †common sites (pediatric [26]; adult [33]).

** Histology listed as 2023 WHO 5th edition/ICC, 2017 WHO 4th revised edition.

Identification of prognostic factors for younger patients after they develop a PTLD is also somewhat difficult. Outcome in many patients appears to correlate with the pathologic diagnosis. Specifically, patients with lesions diagnosed as hyperplasia tend to have a good outcome as lesions often regress following a reduction in immunosuppression or are localized and thus can be removed surgically. In contrast, the lymphomas usually require medical intervention, such as immunotherapy and/or chemotherapy for potential resolution of the disease. However, the polymorphic lesions are less predictable with some studies indicating these lesions are associated with a good prognosis, but other studies showing patients who die of progressive PTLD. Factors such as younger age and lesions located in the head and neck are usually associated with a better prognosis. However, outcome correlations with other factors such as EBV tumor status are not as reliable [25, 26, 27, 28, 38, 39]. Thus, further investigations into PTLD pathogenesis, particularly those lesions arising in young patients, is needed.

## 3 NODULAR LYMPHOCYTE‐PREDOMINANT HODGKIN LYMPHOMA

**FIGURE 1:**
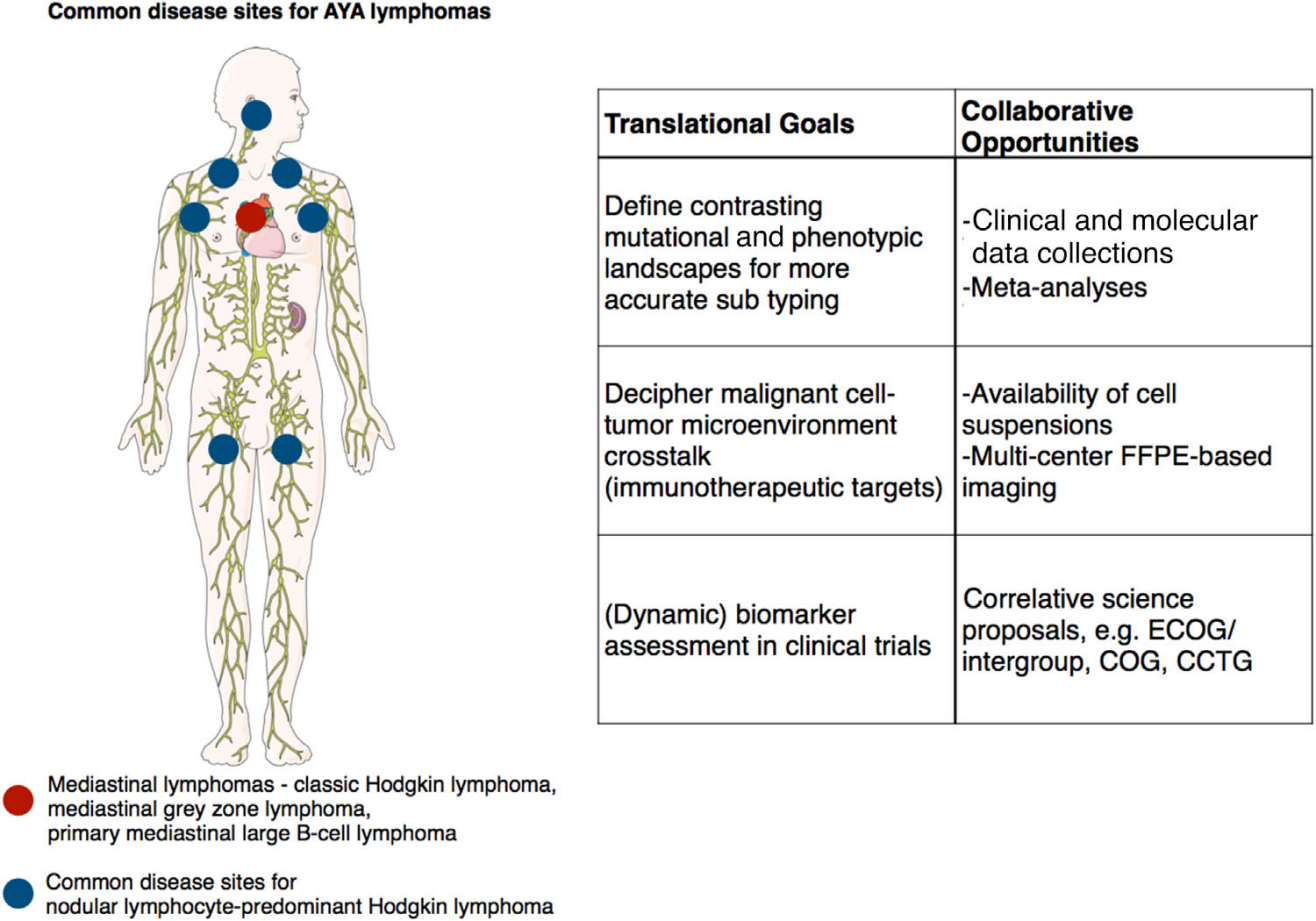
Translational goals and collaborative opportunities in lymphomas that commonly occur in the adolescent and young adult patient population.

Nodular lymphocyte‐predominant Hodgkin lymphoma (NLPHL) is a rare subtype of Hodgkin lymphoma (HL) representing ∼5% of HL cases [21]. The majority of patients with NLPHL are male and diagnosed in the fourth decade of life [40]. While NLPHL has been recognized as a subtype of HL as far back as the Lukes and Butler classification [41], there are many clinical and pathologic features that distinguish it from classic HL (cHL). NLPHL rarely involves the mediastinum, which is a typical disease site for patients with cHL (Figure 1) [42]. Additionally, NLPHL is characterized by multiple relapses occurring even decades after initial diagnosis [43]. Finally, for patients with NLPHL, there is an approximate one percent per year risk of transformation to aggressive large cell lymphoma [44, 45]. Distinct biologic features underly the unique clinical behavior seen in NLPHL.

Similar to the microenvironment for cHL, the malignant lymphocyte‐predominant (LP) cells are surrounded by a vast abundance of immune cells in NLPHL [46]. For NLPHL, the different spatial relationships between the LP cells and the various immune cells have been characterized into six different immunoarchitectural patterns with two typical patterns (A/B) and four variant patterns (C/D/E/F) [47]. Variant patterns represent about a quarter of cases with pattern F being very rarely observed [48]. The variant patterns share a common feature of LP cells residing outside of micronodules suggesting immune evasion [46]. In fact, patterns C/D/E are associated with advanced stage disease at presentation and a higher risk of transformation [45, 48]. Interestingly, CD163 expressing macrophages (consistent with M2 phenotype) have been found in higher abundance for variant pattern E cases providing further molecular evidence of immune suppression and possible mediation of the diffuse malignant involvement in this variant subset [22]. Despite immunoarchitectural patterns having been described two decades ago, robust cell phenotyping has been limited.

Gene expression profiling has shown many transcriptional similarities between Reed‐Sternberg cells of cHL and LP cells such as increased expression of genes within the NF‐κB pathway [49]. However in contrast to Reed‐Sternberg cells, LP cells typically express CD20 and B‐cell transcription factors such as PAX5, OCT2, and BOB1 but rarely CD15/CD30 [46]. For a small subset of NLPHL, LP cells express IgD with association to *Moraxella catarrhalis* suggesting a possible infectious/inflammatory mediated pathogenesis [50]. Based on preliminary reports presented in abstract form and co‐culture experiments, it appears LP cells mediate an environment of immune suppression with expression of PD‐L1 [51, 52]. Further, LP cells are typically surrounded by PD‐1 positive Tfh cell rosettes [46]. The underlying genetic aberrations found in LP cells which may provide insight into lymphomagenesis and the microenvironment composition remain largely unknown.

Genotyping of NLPHL is very challenging due to low tumor cellularity resulting in very low mutation allele frequencies which are often below that of formalin‐fixed paraffin‐embedded artifact [53]. Using fresh frozen samples, Hartmann and colleagues have performed targeted sequencing from pooled LP cells and have identified recurrent mutations in epigenetic modifiers such as *CREBBP* and *EP300* among others similar to many other lymphomas [53]. While genotyping cHL from circulating tumor DNA isolated from patient plasma has emerged as a successful technique [54], preliminary reports for NLPHL suggest plasma allele frequency may be lower than in cHL [55]. Larger studies are needed to evaluate the potential role of ctDNA to genotype NLPHL.

Future efforts are needed to molecularly and genomically characterize NLPHL to develop precision medicine approaches. In an effort to recognize NLPHL as a unique entity, the International Consensus Classification has proposed renaming it to nodular lymphocyte‐predominant B‐cell lymphoma [24]. However, the 5th edition of the WHO classification retains the same name for NLPHL stating further evidence is required prior to implementation of reclassification [22]. Nevertheless, new techniques in molecular biology [52], next generation sequencing [54], and high throughput therapeutic screens [56] will allow for advances in biologically characterizing NLPHL, risk‐stratifying patients, and developing innovative management strategies. International efforts are underway to collaboratively study NLPHL and develop optimal treatments for patients [57].

## 4 FOLLICULAR LYMPHOMA IN YOUNG ADULTS AND PEDIATRIC‐TYPE FOLLICULAR LYMPHOMA

Follicular lymphoma (FL) is the most common indolent type of non‐Hodgkin lymphoma and is characterized by a relapsing and remitting course in most patients. The median age for diagnosis is 61 years [58]. However, FL infrequently arises in younger adults and in the pediatric population, which raises the question of whether there are differences in underlying biological mechanisms and clinical outcomes between younger and older patients. Several retrospective series have reported the outcomes of young adults, defined as less than 40 years old. Gangatharan et al. compared baseline characteristics between young adults and patients between the ages of 40 and 65, finding that both groups had similar clinical characteristics at presentation. However, the 10‐year overall survival was higher in young adults (87% vs. 72%, *p* = 0.029) [59]. Casulo et al. identified over 2500 FL patients in the National LymphoCare Study, of which 6% were 40 years or younger [60]. The findings indicated a favourable outcome for young adults, as the overall survival rate at 8 years was 90%. Furthermore, Conconi et al. described 155 patients younger than 40 years in a multi‐centre, retrospective study, and similarly found excellent outcomes with 10‐year overall survival estimated at 81% [61].

Distinguishing FL arising in young adults from pediatric‐type FL is crucial, as the latter has unique pathological, molecular and clinical correlates. In a series of 23 children reported in 2002, the median age was 11 years and the majority (15/23, 65%) presented with stage I disease [62]. *BCL2* gene rearrangements were uncommon (2/16, 13%) and negative expression of BCL2 by immunohistochemistry was associated with stage I disease and the absence of relapse. Similar findings were later reported by Louissaint *et al.* who confirmed a strong association between young age, stage I disease, high proliferation rate, lack of *BCL2* translocation and long‐term remission [63]. Interestingly, the authors also examined a second series of 58 adult patients with a median age of 57 years (range 18–85) [63] and found that the 13 patients with samples exhibiting a high proliferation rate and lacking the *BCL2* gene rearrangement (median age 37, range 18–61) presented with stage I disease and favourable outcomes, similar to their observations in the pediatric population. It is worth noting that pediatric‐type FL occurs more frequently in males and has a predilection for involving lymph nodes in the head and neck region [62, 63, 64, 65], for reasons that remain unknown. Some debate also remains about whether pediatric‐type FL is part of the same spectrum as pediatric‐type nodal marginal zone lymphoma [66]. Overall, based on the existing evidence, pediatric‐type FL is recognized as a distinct entity in the most recent classification schemes of lymphoid malignancies and needs to be distinguished from other forms such as FL grade 3B (also referred to as follicular large B‐cell lymphoma in the 5th edition of the World Health Organization Classification) [22, 24].

In terms of its genetic landscape, pediatric‐type FL shares frequent *TNFRSF14* alterations (mutations, deletions and loss of heterozygosity of 1p36) with typical FL [67, 68]. Mutations of *IRF8* are also observed in both entities [69, 70, 71]. On the other hand, the mutational landscape of pediatric‐type FL is characterized by lower genomic complexity and lower mutation burden and stands out from typical FL due to the near complete absence of mutations affecting epigenetic modifiers such as *KMT2D*, *CREBPP* or *EZH2*. This observation suggests that unique pathogenetic mechanisms underlie the lymphomagenesis of pediatric‐type FL, which was subsequently demonstrated in studies that revealed recurrent mutations of the *MAP2K1* gene [70, 72]. Similar mutations have been observed in other indolent B‐cell malignancies such as IGHV4‐34‐expressing and variant hairy‐cell leukemia, as well as Langerhans histiocytosis, but are only rarely seen in typical FL [70]. In pediatric‐type FL, *MAP2K1* mutations are seen in 43–49% of cases and are reported as missense mutations localizing to exons 2–3, resulting in downstream ERK pathway activation [68, 72]. Additionally, other MAPK signalling pathway members, including *MAPK1* and *RRAS*, have been reported to be mutated [72, 73], further lending support to the pathophysiological relevance of this pathway in pediatric‐type FL. Taken together, these observations justify the classification of pediatric‐type FL as a distinct entity and the most distinguishing features between pediatric‐type FL and typical FL are summarized in Table 2. Future studies need to address unanswered questions related to this condition, such as the contribution of the tumor microenvironment, genetic predisposition, and any possible immune and/or infectious correlates.

**TABLE 2:** Distinguishing features between pediatric‐type FL and typical FL.

	Pediatric‐type FL	Typical FL
Age	Mostly pediatric but can occur in young adults and even older adults	Adult; median age 61 years, but 6% of patients younger than 40 years
Stage	Limited stage, mostly Ann Arbor stage I	Advanced stage is more frequent than limited stage
Grade	Frequently grade 3A	Grade 1–2 is more common than grade 3A
Proliferation rate	High	Low proliferation rate is typical
*BCL2* rearrangements	Generally absent	Found in ∼85% of cases
Most frequent gene mutations	*TNFRSF14*, *MAP2K1*. Mutations of genes encoding epigenetic modifiers are uncommon.	Most common mutations occur in genes encoding epigenetic modifiers (e.g., *CREBBP*, *KMT2D*, *EZH2*) and in *TNFRSF14*.
Outcome	Relapse is uncommon after treatment.	Relapse is frequent, despite systemic therapy.

## 5 MEDIASTINAL LYMPHOMAS

Primary mediastinal large B‐cell lymphoma (PMBCL), nodular sclerosing cHL, and mediastinal gray zone lymphomas (MGZL), a B‐lymphoid malignancy with features intermediate between cHL and PMBCL, share multiple genetic alterations, phenotypes and clinical characteristics [21]. These include a common early age onset with peak incidences in adolescents and young adults and presentation in the anterior mediastinum. Molecular analyses suggest PMBCL, cHL, and MGZL, also share features of immune escape enabled by somatically acquired mutations [74, 75, 76]. In aggregate, these overlapping characteristics and the common presentation in an anatomically defined space have led to speculation that PMBCL, MGZL and a subset of cHL share histiogenic origins in the thymus warranting further investigation into the group of “mediastinal lymphomas,” although such a grouping is currently not formalized in classification systems [22, 24]. In the following, current knowledge about the pathogenesis of mediastinal lymphomas is reviewed with an emphasis on recent genomics studies and their implications for classification, biomarker development and emergent therapeutic approaches.

Amongst all lymphomas, cHL represents an extreme example in a spectrum of diseases that feature a tumor microenvironment (TME) composed of a multitude of non‐malignant cell types from both the innate and adaptive immune systems. In cHL, these reactive immune cells are believed to be attracted by the malignant Hodgkin Reed‐Sternberg (HRS) cells which are of (post)‐germinal center B‐cell orgin [76]. CHL patients also typically present with disease involving lymph nodes with frequent anterior mediastinal masses [21]. However, exclusive mediastinal disease is rare in CHL. This raises the possibility that malignant or premalignant B cells, derived from nodal germinal centre reactions, may generally migrate secondarily into the thymus during disease progression, although a model of primary thymic B‐cell derivation is also possible in a subset of cases. Molecular hallmarks of the malignant cells include constitutive activation of JAK‐STAT and NFκB signaling [76], and HRS cells frequently harbor multiple mutations that account for their immune escape properties, with the prominent example of gains or amplifications of chromosome 9p24.1 leading to their frequent high expression of PDL1 and/or PDL2 [77]. In cHL, a large number of studies have explored prognostic associations of TME composition with treatment outcome, amongst which the number of tumor‐associated macrophages (TAMs) is the best validated biomarker of poor outcome after standard first and second line therapies [78, 79]. With specific relevance to HL in young patients, a recent study investigating tissue biopsies of pediatric Hodgkin lymphoma patients enrolled in the AHOD0031 trial found significant associations of tumor microenvironment components in the TME and TARC expression with poor treatment outcome, and the investigators harnesses this information for prognostic biomarker development (PHL‐9C assay) [80].

PMBCL is an aggressive lymphoma presumed to arise from thymic medullary B‐cells [81]. However, immunophenotyping and mutational data have also suggested that PMBCL might be derived from germinal centre B cells [82], and it remains unresolved as to how this putative histiogenic thymic B‐cell origin can be reconciled with the molecular marks of germinal centre transition [83]. PMBCL accounts for approximately 2% of all NHL cases occurring mostly in young female adults and, based on pure histologic criteria, would be classified as a variant form of diffuse large B cell lymphoma (DLBCL). However, PMBCLs exhibit distinct clinical and immunohistochemical features compared to nodal DLBCLs, and mutational analysis and gene expression profiling has established PMBCL as a related entity of CHL [75, 84]. PMBCL also shares several main molecular hallmarks with CHL; including a high constitutive activation of JAK‐STAT and NFκB signaling [85], as well as immune privilege features vested in somatic mutations, for example, *B2M*, *CIITA*, and *CD58* [86]. Differentiation of PMBCL from DLBCL can be difficult by clinic‐pathological consensus procedures for diagnostic purposes, and a recently developed digital gene expression profiling‐based assay, including the assessment of 30 genes (Lymph3CX), can aid in making an accurate diagnosis of PMBCL [87]. Genomic profiling studies of PMBCL confirmed the distinct mutational pattern in contrast to DLBCL which might provide additional avenues for diagnostic assay development.

MGZL is a B‐cell lymphoma with overlapping clinical, morphological, phenotypic and molecular features intermediate between PMBCL and nodular sclerosis CHL [21]. MGZL typically presents as a localized anterior mediastinal mass with frequent supraclavicular lymph nodes and lung or pleural extension [74]. As for PMBCL and CHL, MGZL also frequently presents in young patients, more often in males. Exclusive mutational features of MGZL have not been described, and MGZL shares genomic variants with CHL and PMBCL that alter the JAK‐STAT, NFκB and nuclear transport pathways with absent *BCL2* or *BCL6* rearrangements [74, 88, 89]. Information on the tumor microenvironment of MGZL is sparse with two gene expression profiling‐based studies demonstrating variable immune cell composition [90, 91]. One of these studies suggested a higher abundance of macrophages than is seen in CHL and PMBCL [90].

The importance of the TME in all mediastinal lymphoma entities has suggested that further elucidation of the relevant mechanisms involved in cellular crosstalk might be therapeutically exploited by appropriate targeting of the responsible tumor‐immune cell interactions occurring within the TME [92]. Indeed, this concept is already supported by the results of a number of immunotherapy approaches, a prominent example being the very high objective response rates obtained using PD‐1 checkpoint inhibitors in CHL and PMBCL [93, 94].

## 6 FUTURE PERSPECTIVE

As illustrated in the disease‐specific pathogenesis reviews above, the lymphoma field is progressing towards improved molecular disease taxonomies that provide the foundation for clinical decision making and future incorporation of novel targeted agents and immunotherapeutic approaches through clinical trial testing. These dynamic taxonomies are in part reflected in the current classification systems of the WHO and ICC [22, 24] with prominent examples in PTDL, HL, FL, and mediastinal lymphomas (incl. MGZL) as discussed in this review.

However, the improving knowledge about general pathogenesis of specific lymphoma entities often does not reflect the specific biology of lymphomas in the AYA population. More explicitly, with few notable exceptions, the contrasting biology and outcomes of lymphomas across the full age range are not studied and the AYA population is typically only represented as edge cases in standard of care real‐world studies and clinical trials using either pediatric or adult oncology treatment protocols. Therefore, it follows logically that systematic collaboration between the separate fields of pediatric and adult oncology practice must be encouraged with respect to comprehensive and inclusive biobanking, joint clinical trial design, clinical data collections and sharing of, for example, genomics datasets. These considerations also apply to the integration of diagnostic disciplines into research studies, that is, pathology and radiology, with the intention to remove biospecimen and data silos between the pediatric versus adult oncology fields, and clinical trials groups in particular. Since many lymphomas are relatively rare in the AYA population, the removal of institutional and field‐specific barriers will be critical to enable meaningful and statistically powered analysis in joint study designs that are associated with high‐quality data repositories. In particular, for the construction of comprehensive datasets, future efforts will include the need for data collection of race/ethnicity, including disaggregated population subgroups, and social factors that may impact disease biology, occurrence and outcomes. Linkage of registry data with other claims data may be valuable for advancing population research and identifying novel research directions, rather than simply applying findings from adult studies and clinical practice to younger patients.

The main translational goals and related opportunities for interdisciplinary collaboration are illustrated in Figure 1 using the example of NLPHL and mediastinal lymphomas. Importantly, a better biological understanding of the various diseases and thus improved treatment of AYA lymphomas will require the close cooperation of pediatric and adult cooperative groups. We anticipate that the establishment of an AYA consortium, in which ‘Disease biology, Etiology and Diagnosis’ will feature as a valuable platform for thought and data exchange, will serve as a critical catalyst for future collaborative efforts with the goal to improve outcomes and quality of life for AYA patients.

## AUTHOR CONTRIBUTIONS

All authors wrote and approved the manuscript.

## CONFLICT OF INTEREST STATEMENT

The authors declare no conflict of interest.

## FUNDING INFORMATION

The authors received no specific funding for this work.

## ETHICS STATEMENT

The authors have confirmed ethical approval statement is not needed for this submission.

## PATIENT CONSENT STATEMENT

Author has confirmed patient consent statement is not needed for this submission.
